# Pneumobilia and Hepatic Abscess Secondary to Polymicrobial Infection of the Biliary Tree

**DOI:** 10.7759/cureus.101273

**Published:** 2026-01-11

**Authors:** Sunandan Bhattacharya, Darshana Wickramasinghe, Thaw Myint Thu, Prakash Velmurugan, Binod Bekoju

**Affiliations:** 1 Acute Medicine, General Internal Medicine, Medway NHS Foundation Trust, Chatham, GBR; 2 Microbiology, Medway NHS Foundation Trust, Chatham, GBR; 3 Cardiology, Medway Maritime Hospital, Gillingham, GBR; 4 Internal Medicine, Medway NHS Foundation Trust, Chatham, GBR; 5 Family Medicine, Medway NHS Foundation Trust, Chatham, GBR

**Keywords:** antibiotics, cholangitis, follow up imaging, hepatic abcess, klebsiella pneumonia, novel organism, pneumobilia, prolonged antibiotics, sepsis, streptococcus anginosus

## Abstract

A 76-year-old man with a history of choledocho-lithiasis and cholecystectomy presented with recurrent epigastric pain, fever, and elevated infection markers. He was initially managed as a case of possible ascending cholangitis with oral antibiotics, which failed to treat the infection. Due to clinical deterioration, a CT abdomen and pelvis was arranged to investigate the aetiology. The CT images revealed pneumobilia, signs of cholangitis with intrahepatic duct dilatation, and evidence of a liver abscess. His blood cultures grew *Streptococcus constellatus*, *Streptococcus anginosus*, and *Klebsiella pneumoniae*. Subsequent treatment with IV ceftriaxone and metronidazole led to resolution of the pneumobilia and hepatic abscess formation on follow-up MRI liver scan. This suggests that the pneumobilia likely persisted due to gas-forming bacteria, particularly *Klebsiella pneumoniae*. The persistence of *Streptococcus anginosus* highlights the need for prolonged, sensitivity-based antibiotic therapy to fully eradicate virulent biliary infections and prevent complications such as hepatobiliary abscess. Follow-up radiological investigation to check resolution of pneumobilia and cholangitis post treatment with targeted antibiotic therapy is crucial to prevent complications, and if there is persistence of radiological evidence indicating infective changes, the antibiotic course should be prolonged.

## Introduction

Pneumobilia, the presence of gas within the biliary tree, can arise from various aetiologies. While often a benign consequence of prior surgical interventions such as endoscopic retrograde cholangiopancreatography (ERCP) or laparoscopic cholecystectomy, its presence can also signal significant underlying pathology, including biliary enteric fistulas or, less commonly, gas-forming bacterial infections of the biliary system [1].

Bacterial cholangitis, an infection of the bile ducts, is a significant cause of morbidity and mortality. The involvement of gas-forming bacteria, particularly *Klebsiella pneumoniae*, is notable due to its potential to produce intraluminal gas and its association with more severe disease presentations, including liver abscess formation [2]. Furthermore, the *Streptococcus anginosus* group, including *S. constellatus* and *S. anginosus*, is increasingly recognised for its highly virulent and indolent nature, often leading to deep-seated infections and abscesses, necessitating prolonged and targeted antibiotic therapy [3].

This case report aims to underscore the importance of considering significant infectious aetiologies for pneumobilia, especially in patients with a history of biliary interventions and recurrent symptoms. It highlights a unique presentation in which pneumobilia, initially thought to be benign post-procedural, was ultimately attributed to polymicrobial gas-forming bacterial cholangitis, emphasising the diagnostic challenge and the critical need for comprehensive microbiological identification and prolonged, sensitivity-based antibiotic treatment to prevent severe complications.

## Case presentation

A 76-year-old man who had previously been diagnosed with choledocholithiasis and managed with ERCP, as well as subsequent laparoscopic cholecystectomy one and a half years previously, presented with on-and-off epigastric pain and fever to an ambulatory acute medicine clinic. He had no other diagnosed medical conditions and was known to have penicillin intolerance. He had, in fact, presented to the emergency department with similar symptoms four days prior and was discharged home with a diagnosis of gastritis.

**Figure 1:**
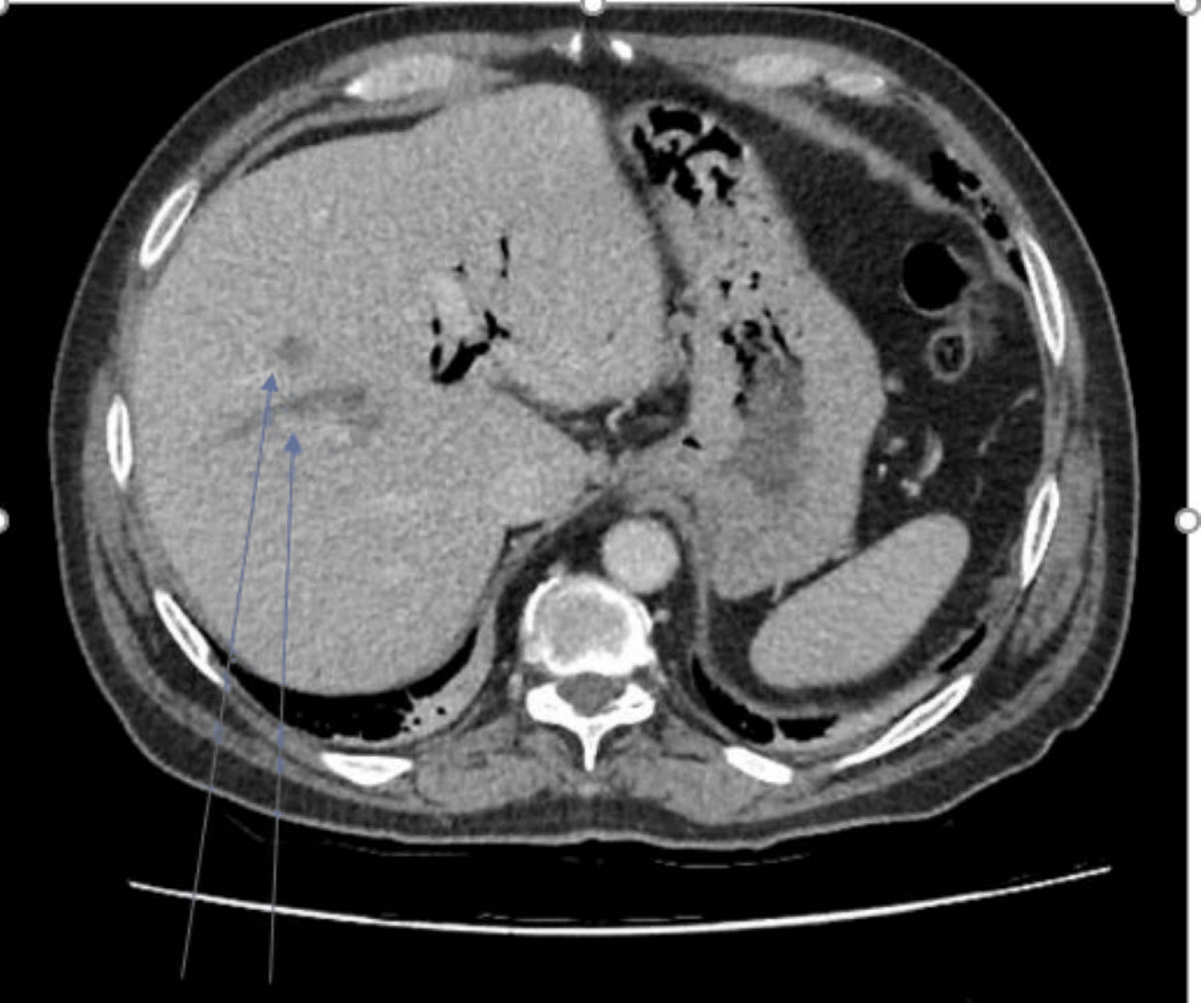
Initial CT abdomen and pelvis with hypodense liver lesions likely suggestive hepatic abcess

He was noted to have raised infection markers, with white cell counts of 14 × 10⁹/L and a C-reactive protein (CRP) of 12.5, and deranged liver function tests, including an alanine aminotransferase (ALP) of 236, alkaline phosphatase (ALT) of 66, and total bilirubin of 12 (Table 1). He was managed conservatively with oral levofloxacin and metronidazole. Blood cultures were sent, and an outpatient CT abdomen was arranged. However, he clinically deteriorated with persistent fever and worsening abdominal pain and required readmission two days after discharge from the ambulatory care unit. Blood tests on repeat admission showed a significantly elevated CRP of 349 and a white cell count of 14.3 × 10⁹/L (Table 1). His blood cultures grew *Streptococcus constellatus*, *Streptococcus anginosus*, and *Klebsiella pneumoniae*, which were sensitive to co-amoxiclav, gentamicin, and ciprofloxacin. He was commenced on treatment with IV ceftriaxone and metronidazole after discussion with the local microbiology team. This choice of antibiotics was made as he was penicillin intolerant, did not show any signs of improvement after oral antibiotic therapy, and due to the polymicrobial nature of the infection. The CT abdomen and pelvis (Figure 1) showed evidence of new pneumobilia, as well as signs of infection of the biliary tree with intrahepatic duct dilatation and several hypodense liver lesions suggestive of hepatic abscesses. There were no signs of microperforation, extraluminal gas bubbles, or pericolic fat stranding to demonstrate an alternative source of polymicrobial flora that could result in hepatic abscess formation. He responded well to this antibiotic regimen, clinically improved, and was discharged after four days of inpatient IV antibiotic therapy, with a plan for continued monitoring and continuation of IV ceftriaxone and PO metronidazole to complete a six-week course in a virtual ward. 

**Table 1:** Blood tests including inflammatory markers and liver function tests with dates ^#^Values above the reference range. ^$^Values below the reference range. AKI: acute kidney injury, ALT: alanine aminotransferase, GFR: glomerular filtration rate, CRP: C-reactive protein, MCH: mean corpuscular haemoglobin, MCHC: mean corpuscular haemoglobin concentration, MCV: mean corpuscular volume, RBC: red blood cell, RDW: red cell distribution width, WBC: white blood cell.

Parameters	24-03-2024	26-03-2024	15-04-2024	29-04-2024	Units	Range
AKI stage	0	0	0	0		
Albumin	43	41	39	38	g/L	35-50
Alkaline phosphatase	236^#^	197^#^	332^#^	125	U/L	30-130
ALT	66^#^	73^#^	38	45	U/L	<50
Creatinine	75	86	78	78	umol/L	59-104
Estimated GFR	88	75	84	84	mL/min/1.73m^2^	>90
Haemoglobin	134	138	130	128	g/L	130-170
CRP	12.5^#^	349.0^#^	2.6	0.8	mg/L	<4
Haematocrit	0.40	0.41	0.40	0.39	L/L	0.40-0.50
Potassium	3.3^$^	3.3^$^	4.4	3.5	mmol/L	3.5-5.3
Automated lymphocyte count	0.6^$^	0.5^$^	2.5	1.6	× 10^9^/L	1.0-4.0
MCH	30.1	30.3	29.3	29.8	pg	27.0-34.0
MCHC	339	338	327	331	g/L	315-345
MCV	88.7	89.5	89.6	90	fL	80.0-100.0
Automated monocyte count	1.0	0.7	0.5	0.3	× 10^9^/L	<1.0
Sodium	139	134	137	141	mmol/L	133-146
Automated neutrophil count	12.4^#^	13.0^#^	3.9	3.8	× 10^9^/L	2.0-7.0
Platelet count	288	219	468^#^	225	× 10^9^/L	150-410
RBC	4.45^$^	4.54	4.43^$^	4.29	× 10^12^/L	4.50-5.50
RDW	13.0	12.8	13.4	13.8	%	8.0-16.0
Total bilirubin	12	29^#^	7	6	umol/L	0-21
Urea	3.7	4.1	3.3	3.2	mmol/L	2.5-7.8
WBC	14	14.3^#^	7.3	6.2	× 10^9^/L	4.0-11.0

**Figure 2:**
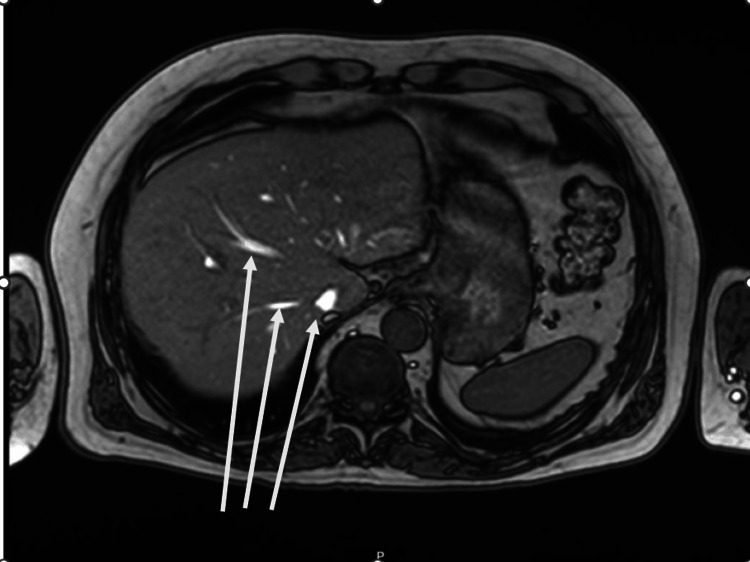
MRI with signs of cholangitis and resolving abcess

An MRI liver scan (Figure 2), which was performed two weeks post discharge, showed cholangitis with a resolving abscess and complete resolution of pneumobilia.

His blood tests, repeated weekly during the next six weeks, revealed progressive improvement in infection markers and liver function tests. A follow-up ultrasound of the abdomen was performed one month after completion of antibiotic therapy and did not show any remaining evidence of cholangitis. He has since been well and has not had any recurrence of symptoms.

## Discussion

This case report highlights a unique instance of pneumobilia in a patient with a history of biliary interventions, ultimately attributed to polymicrobial bacterial cholangitis caused by gas-forming *Klebsiella pneumoniae* and the virulent *Streptococcus anginosus* group. This presentation underscores the diagnostic challenge of pneumobilia, which, while often benign post-procedurally, necessitates thorough investigation with radiological examination when associated with recurrent symptoms and infection [1].

Pneumobilia is commonly iatrogenic or due to biliary enteric fistulas. However, its persistence might signify colonisation or infection with gas-forming bacteria, which may lead to severe cholangitis or liver abscess if left untreated. In our patient, the initial broad-spectrum oral antibiotics failed to provide sufficient cover, and the patient required readmission for IV antibiotic therapy. Symptom recurrence and positive blood cultures for *Klebsiella pneumoniae*, *Streptococcus constellatus*, and *Streptococcus anginosus* strongly implicated an infectious aetiology. The complete resolution of pneumobilia on MRI after targeted antibiotic therapy further supports this, distinguishing it from other causes that typically result in persistent gas within the biliary tree.

*Klebsiella pneumoniae* is a well-documented gas-forming organism causing severe intra-abdominal infections, including liver abscesses and cholangitis, with reported cases of pneumobilia directly attributed to it [2]. Similarly, the *Streptococcus anginosus* group is known for causing deep-seated, often indolent, abscess-forming infections [3]. Their isolation in blood cultures, coupled with recurrent symptoms and abscess formation, emphasises their pathogenic potential and the need for prolonged, sensitivity-based antibiotic courses to prevent relapse.

This case serves as a reminder for clinicians to meticulously evaluate pneumobilia, especially in the presence of recurrent symptoms, and to consider gas-forming bacterial cholangitis. Prompt microbiological identification and tailored, prolonged antibiotic therapy are paramount to ensure complete eradication and prevent complications. Furthermore, there is a need for follow-up radiological imaging to assess resolution of pneumobilia and cholangitis, thereby preventing complications such as abscess formation.

## Conclusions

In patients with a history of biliary interventions and recurrent symptoms of abdominal pain and fever with raised infection markers, pneumobilia should prompt thorough investigation for active infection, particularly gas-forming bacterial cholangitis, rather than being solely attributed to benign post-procedural changes. Recurrent biliary symptoms associated with pneumobilia necessitate comprehensive microbiological evaluation, including blood cultures, to identify specific causative organisms, especially virulent and gas-forming bacteria.

Resolution of pneumobilia and cholangitis on follow-up imaging with CT, MRI, or ultrasound of the abdomen after appropriate antibiotic treatment can serve as a valuable indicator of successful infection eradication. If infectious changes persist, the course of antibiotics should be extended appropriately. However, follow-up imaging should always be undertaken in conjunction with blood tests to assess for ongoing active infection following completion of antibiotic therapy.
